# Retention and viral suppression in a cohort of HIV patients on antiretroviral therapy in Zambia: Regionally representative estimates using a multistage-sampling-based approach

**DOI:** 10.1371/journal.pmed.1002811

**Published:** 2019-05-31

**Authors:** Izukanji Sikazwe, Ingrid Eshun-Wilson, Kombatende Sikombe, Nancy Czaicki, Paul Somwe, Aaloke Mody, Sandra Simbeza, David V. Glidden, Elizabeth Chizema, Lloyd B. Mulenga, Nancy Padian, Chris J. Duncombe, Carolyn Bolton-Moore, Laura K. Beres, Charles B. Holmes, Elvin Geng

**Affiliations:** 1 Centre for Infectious Disease Research in Zambia, Lusaka, Zambia; 2 University of California, San Francisco, San Francisco, California, United States of America; 3 Ministry of Health, Lusaka, Zambia; 4 University of California, Berkeley, Berkeley, California, United States of America; 5 International Association of Providers of AIDS Care, Washington, District of Columbia, United States of America; 6 University of Alabama at Birmingham, Birmingham, Alabama, United States of America; 7 Johns Hopkins University, Baltimore, Maryland, United States of America; 8 Georgetown University, Washington, District of Columbia, United States of America; University of Southampton, UNITED KINGDOM

## Abstract

**Background:**

Although the success of HIV treatment programs depends on retention and viral suppression, routine program monitoring of these outcomes may be incomplete. We used data from the national electronic medical record (EMR) system in Zambia to enumerate a large and regionally representative cohort of patients on treatment. We traced a random sample with unknown outcomes (lost to follow-up) to document true care status and HIV RNA levels.

**Methods and findings:**

On 31 July 2015, we selected facilities from 4 provinces in 12 joint strata defined by facility type and province with probability proportional to size. In each facility, we enumerated adults with at least 1 clinical encounter after treatment initiation in the previous 24 months. From this cohort, we identified lost-to-follow-up patients (defined as 90 or more days late for their last appointment), selected a random sample, and intensively reviewed their records and traced them via phone calls and in-person visits in the community. In 1 of 4 provinces, we also collected dried blood spots (DBSs) for plasma HIV RNA testing. We used inverse probability weights to incorporate sampling outcomes into Aalen–Johansen and Cox proportional hazards regression to estimate retention and viremia. We used a bias analysis approach to correct for the known inaccuracy of plasma HIV RNA levels obtained from DBSs. From a total of 64 facilities with 165,464 adults on ART, we selected 32 facilities with 104,966 patients, of whom 17,602 (17%) were lost to follow-up: Those lost to follow-up had median age 36 years, 60% were female (*N* = 11,241), they had median enrollment CD4 count of 220 cells/μl, and 38% had WHO stage 1 clinical disease (*N* = 10,690). We traced 2,892 (16%) and found updated outcomes for 2,163 (75%): 412 (19%) had died, 836 (39%) were alive and in care at their original clinic, 457 (21%) had transferred to a new clinic, 255 (12%) were alive and out of care, and 203 (9%) were alive but we were unable to determine care status. Estimates using data from the EMR only suggested that 42.7% (95% CI 38.0%–47.1%) of new ART starters and 72.3% (95% CI 71.8%–73.0%) of all ART users were retained at 2 years. After incorporating updated data through tracing, we found that 77.3% (95% CI 70.5%–84.0%) of new initiates and 91.2% (95% CI 90.5%–91.8%) of all ART users were retained (at original clinic or transferred), indicating that routine program data underestimated retention in care markedly. In Lusaka Province, HIV RNA levels greater than or equal to 1,000 copies/ml were present in 18.1% (95% CI 14.0%–22.3%) of patients in care, 71.3% (95% CI 58.2%–84.4%) of lost patients, and 24.7% (95% CI 21.0%–29.3%). The main study limitations were imperfect response rates and the use of self-reported care status.

**Conclusions:**

In this region of Zambia, routine program data underestimated retention, and the point prevalence of unsuppressed HIV RNA was high when lost patients were accounted for. Viremia was prevalent among patients who unofficially transferred: Sustained engagement remains a challenge among HIV patients in Zambia, and targeted sampling is an effective strategy to identify such gaps in the care cascade and monitor programmatic progress.

## Abstract 2

Izukanji Sikazwe and co-workers report on the use of routine program data to estimate retention of patients in HIV care in Zambia.

## Author summary

**Why was this study done?:**

- Retention and HIV RNA suppression in HIV treatment programs represent critical metrics of success, but regionally representative estimates in longitudinal cohorts remain uncommon.
- Most treatment programs, whether at the national or sub-national level, lack data systems able to capture patient movement across facilities, which may lead to underestimates of retention.
- HIV RNA suppression levels from routine program monitoring or large-scale cross-sectional studies may miss patients who are lost to follow-up, and therefore those who had been on treatment, thus underestimating the prevalence of viremia.
- Intensive ascertainment of care status and HIV RNA levels in a numerically small but randomly selected sample of patients with unknown outcomes can improve our understanding of treatment success in real-world program settings.

**What did the researchers do and find?:**

- We used a multistage sampling approach, in which we first selected facilities and then, within each facility, selected a random sample of patients who were lost to follow-up—defined as no contact with a health facility for 90 or more days after last their missed appointment—for intensive tracing. We also collected dried blood spots in Lusaka Province to determine viral load levels in both a sample of lost patients and in-care patients to estimate the prevalence of viremia in both populations.
- We found that among 165,464 patients on treatment in 64 facilities, 28,111 (17%) were lost to follow-up. We traced 2,892 of the lost (16%): and found 412 (14%) had died and 1,751 (61%) remained alive. Of those alive, 1,293 (74%) continued to receive treatment, 255 (15%) had stopped, and care status could not be determined in 12%.
- Among all ART patients, using data known to the program before tracing, retention was 67.7% at 2 years; after incorporating findings among the lost patients, retention was 91.2%.
- Among 1,044 participants with a viral load determination (901 in care and 143 who were lost), viremia was present in 18.1% of those in care, 71.3% of those lost to follow-up (49.8% of those lost and in care elsewhere and 83.9% of those lost and not in care), and 24.8% overall.

**What do these findings mean?:**

- We found that patient retention in public ART facilities in Zambia was higher than apparent in data collected during routine care and monitoring.
- Estimates of viremia that do not account for elevated levels in patients who stop treatment (and are lost to follow-up from cohort studies) or are missing from cross-sectional studies may overestimate treatment success.
- Viremia among patients lost from one facility who reported engagement in a new facility was markedly higher than among patients who remained engaged in their original facility: Even though durable discontinuation from care was relatively infrequent, strategies to consistently engage patients to enhance retention and viral suppression are urgently needed.

## Introduction

Assessments of retention and HIV RNA suppression levels after HIV treatment initiation in routine program settings represent the backbone of data-driven public health efforts to bring the epidemic under control. As HIV treatment reaches more patients in the era of test-and-treat, the remaining gaps in the cascade are likely to shift toward retention and adherence as the key modifiable mediators of success [1–3], which warrant careful assessment. Identifying when and why patients miss clinical visits, fail to pick up medications, and become viremic can help programs focus attention on vulnerable periods [4]. In addition, identifying facilities where retention and viral suppression are lower than at other similar settings can also direct targeting of additional health systems investments. Indeed, at this phase of the HIV treatment response, relatively widespread geographical access to treatment and large numbers on treatment already mean that the next phase in improvement efforts should focus on retention and suppression.

The importance of accurate measures of retention and viral suppression in routine care delivery settings, however, brings critical challenges in monitoring into focus. First, many patients move for social or livelihood reasons. Most programs lack data systems that are integrated in a region to capture movement across facilities. Second, even networked systems will not link records when patients enroll in new facilities using different names or identifiers, which is common to avoid being considered uncommitted patients by healthcare workers. In either case, routinely available data may underestimate retention [5,6]. Similarly, routine clinic-based viral load monitoring will fail to account for patients who are not coming back to clinic (i.e., lost to follow-up). In an analysis from the International Epidemiology Databases to Evaluate AIDS (IeDEA), investigators found that 94% of retained patients were virally suppressed, but this figure dropped to 45% when all lost patients were assumed to be viremic [7]. The Zambia Population-based HIV Impact Assessment (ZAMPHIA) suggested viral suppression in nearly 90% of people self-reporting ART use; patients lost to follow-up from treatment programs (and who have not been on treatment for some time) may not be captured in the denominator, thus potentially overestimating suppression [8].

In this study, we examined retention and viral suppression in a large public health program across 4 provinces in Zambia, a country with an estimated 1,200,000 adults living with HIV [8–10]. Building on previous work, we used a sampling-based approach in which we first selected facilities from 4 provinces (with probability proportional to facility size) and then intensively tracked a random sample of individuals (inversely proportional to facility size) lost to follow-up in each of these selected sites. In addition, in 1 of the 4 provinces (Lusaka), we assessed data on plasma HIV RNA suppression levels among a sample of both in-care and lost-to-follow-up patients. This approach yielded both a representative estimate of overall retention and viral suppression in a large region of Zambia and site-level estimates of retention with enough precision to assess site-to-site variation [11].

## Methods

### Ethical approval

The protocol and study were approved by the University of Zambia Biomedical Research Ethics Committee (004-06-14), and the institutional review board of the University of Alabama at Birmingham School of Medicine (F160122006). The full analysis protocol is available in S1 Appendix. The study adhered to good practice guidelines for reporting for cohort studies as presented in the STROBE statement (S2 Appendix).

### Patients and sampling

Our sampling frame consisted of HIV-positive adults 18 years or older who sought HIV care and treatment services during a 24-month period (1 August 2013 to 31 July 2015) across 64 public health facilities supported with funding from the US President’s Emergency Plan for AIDS Relief/Centers for Disease Control and Prevention through the Centre for Infectious Disease Research in Zambia (CIDRZ) in 4 provinces (Western, Lusaka, Eastern, and Southern) in Zambia. We used a multistage-sampling-based approach to obtain corrected estimates of retention and viremia [12]. Briefly, we stratified 64 total facilities by province (Eastern, Western, Southern, and Lusaka) and facility type (hospital, urban health center, and rural health center) and selected facilities within these 12 joint strata with probability proportional to size. In each selected facility, we enumerated those lost to follow-up (defined as at least 90 days late for the last visit and not documented to have died or transferred out according to the electronic medical record [EMR] system), and selected a random sample with a sampling probability inversely proportional to facility size. In 14 Lusaka facilities selected for this study, we obtained dried blood spot (DBS) samples for determining plasma HIV RNA level (viral load) from both lost patients as well as a systematic sample of patients (defined as every 10th patient) retained at the facility (S1 Fig). This study predated routine plasma HIV RNA monitoring for treatment.

### Procedures and measurements

Data about patient appointments and visits and sociodemographic and clinical characteristics were obtained from the EMR system in Zambia (SmartCare) and used to enumerate the lost-to-follow-up patients. Lost patients were traced between October 2015 and June 2016 by chart review, phone calls, and in-person visits within the community. We recruited peer health workers with in-depth knowledge of patient flow within facilities and familiarity with the surrounding communities to carry out tracing. Patients were classified as died if review of EMR, paper records, or the tracing process found evidence that the patient was deceased. Patients were classified as alive if spoken to in person or an informant was contacted and reported knowledge of the patient but no knowledge of death. When information about a patient was collected from more than 1 informant and was discordant, we used information from closer relations (e.g., we prioritized information from a spouse over that from a neighbor). When lost patients were contacted in person, we asked, “Have you seen any doctor, nurse or other professional health worker (like, pharmacist) for treatment of HIV since your last visit which we have on file, which was on [*X* date] at the [original clinic]?” and recorded the date of that subsequent visit if the answer was yes (S3 Appendix). Current care status (i.e., retained in care) was established only if found through chart review or the patient was contacted in person. Identities were established by name, nicknames, age, occupation, height, sex, and location of residence. In Lusaka, we trained tracers to collect DBS, assess sample quality, and transport DBS cards to the CIDRZ central laboratory. We used the AmpliPrep/COBAS TaqMan HIV-1 Test, version 2.0, to quantify human immunodeficiency virus type 1 (HIV-1) RNA in DBSs. Viral suppression was defined as less than 1,000 copies/ml.

### Analyses

We determined “naïve” estimates of retention using only data available from the facility EMR for the entire cohort of ART users (which included all patients on ART in the 2-year period of observation, 1 August 2013 to 31 July 2015) as well as for new ART initiators (who started ART in this 2-year window). We carried out “revised” estimates that incorporated tracing outcomes through use of probability weights [13]. Weights were inverse to the probability of selection at both the patient and facility level, a process that seeks to yield regionally representative estimates [13,14] (S2 Fig). In the naïve analysis, we estimated the prevalence of 4 care states during the 2-year observation period using the Aalen–Johansen method [15]: (1) alive and in care at original clinic, (2) transferred to a new facility (which included only official transfers), (3) lost to follow-up, or (4) died. In revised estimates, after incorporating findings from tracing through probability weights, we estimated the prevalence of patients over time in the following 4 states: (1) alive and in care at the original clinic, (2) transferred to a new clinic (which included both official and unofficial transfers), (3) alive but out of care, or (4) died. We used Cox proportional hazards models to identify characteristics associated with being out of care or deceased in the revised estimates. We examined the proportional hazards assumption using Schoenfeld residuals [16] (S1 Table), and, in addition to the application of inverse probability weights to account for sampling, we used inverse probability weights to address missing predictor data for CD4 count, WHO stage, marital status, and level of education [17]. We used robust variance estimates to account for clustering by clinic.

In Lusaka Province, we estimated the prevalence of viremia (viral load ≥ 1,000 copies/ml) among lost patients alone, then among patients in care at their original clinic, and finally overall (combining both) by applying sampling weights. We managed bias incurred via the inaccuracy of DBS-based viral load results by using the documented sensitivity of 80.8% and specificity of 87.3% (for detecting a viral load of ≥1,000 copies/ml) as compared to plasma HIV RNA determination through an outcome misclassification correction approach [18,19] (S4 Appendix). We used inverse probability weights to account for sampling (S1 Fig) and missing data in all analyses [17]. Post hoc analyses not predefined in the protocol include analyses restricted to the contemporary cohort of those initiating ART on or after 1 August 2013, analyses using DBS viral load outcome misclassification correction methods, and an analysis of predictors of viremia.

## Results

**Fig 1:**
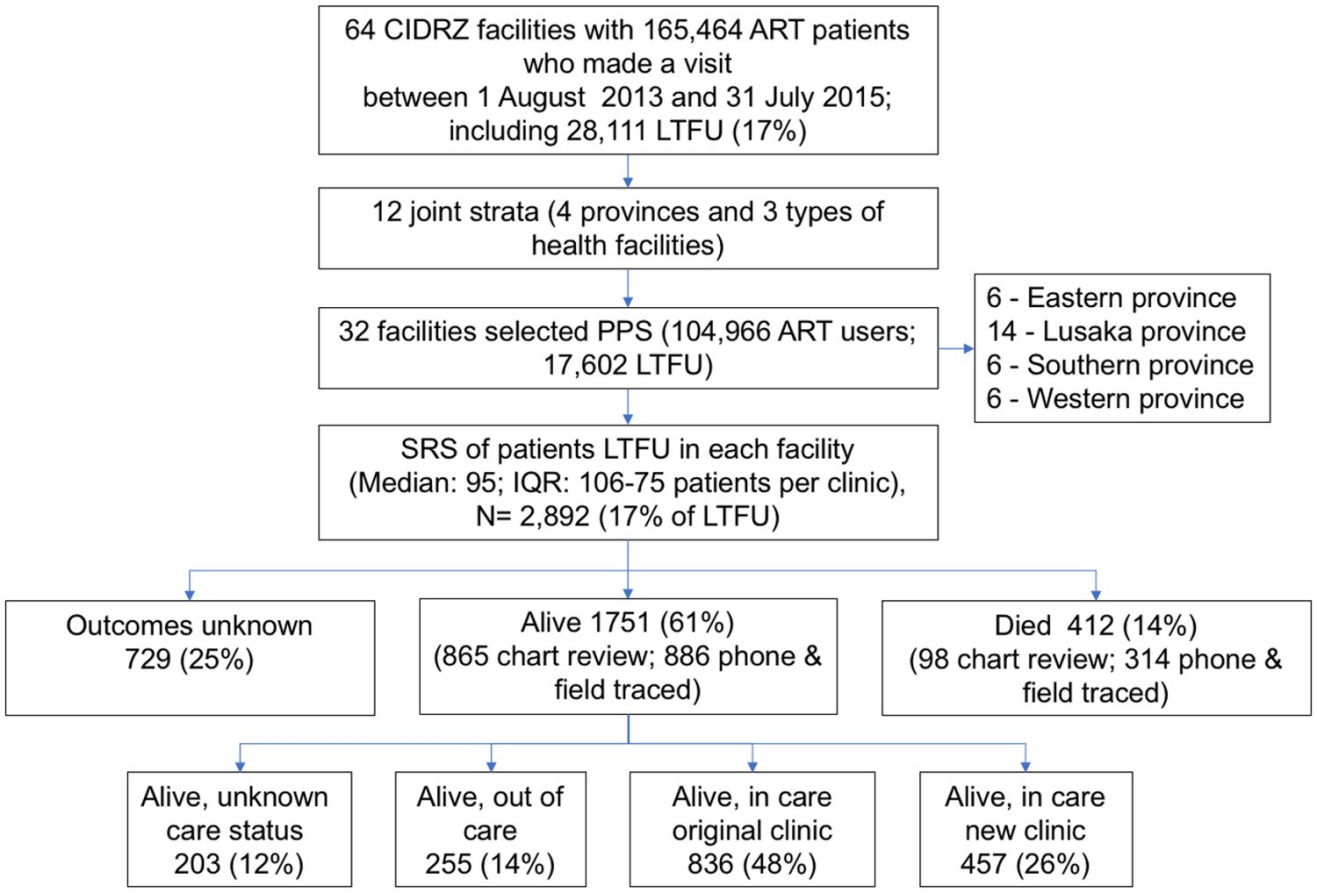
Flowchart depicting sampling and patient outcomes. CIDRZ, Centre for Infectious Disease Research in Zambia; LTFU, lost to follow-up; PPS, probability proportional to size; SRS, simple random sample.

As described in previous work, 165,464 patients on ART had at least 1 encounter in the 64 health facilities over the 24 months between 1 August 2013 and 31 July 2015 (Fig 1) [12], of whom 28,111 (17%) were considered lost to follow-up at the time of sampling. At the 32 selected sites, 104,966 patients made any visit during that time, and 17,602 (17%) were lost to follow-up. We selected a random sample of 2,892 lost patients (16% of 17,602 lost patients at 32 selected facilities and 10% of all 28,111 lost in all 64 facilities) for intensive tracing to ascertain current care status. Of the 2,892 lost and traced, updated information was found for 2,163 (75%), of whom 1,751 (81%) were alive. Among those found alive, 836 (48%) were still in care at the original health facility, 457 (26%) had transferred to another facility, and 255 (15%) were out of care; for 203 (12%) care status remained undetermined (Fig 1). Patient characteristics among those patients lost, traced, and for whom updated care status was ascertained were similar to those of the overall population of lost patients in the total ART cohort (Table 1). Compared to the total ART cohort, new ART initiates with updated care status (*N* = 483) had a shorter duration of ART (88 days; IQR 1–224), mostly enrolled in care between 2013 and 2015 (85%; the other 15% enrolled before 2013, but started ART in the 2-year observation period), and appeared younger in age (median 33 years; IQR 28–40) (S2 Table).

**Table 1:** Characteristics of all patients at start of observation on 31 August 2013 (*N* = 165,464).

Characteristic	Total ART cohort	Lost	Sampled	Successfully traced	Alive	Updated care status
**Total number**	165,464	28,111	2,892	2,163	1,751	1,548
**Age at last visit (years)**	39 (33–34)	36 (30–43)	37 (31–44)	37 (31–44)	37 (31–43)	37 (31–44)
**Male**	59,719 (36)	11,241 (40)	1,187 (41)	909 (42)	703 (40)	615 (40)
**Enrollment CD4 count (cells/μl)**^a^	224 (119–357)	220 (115–354)	220 (111–362)	217 (112–352)	231 (124–370)	230 (120–372)
**ART initiation CD4 count (cells/μl)**^b^	201 (111–312)	201 (108–318)	200 (103–313)	199 (105–309)	210 (115–319)	208 (114–321)
**WHO stage at enrollment**						
Stage 1	62,116 (38)	10,690 (38)	1,059 (37)	783 (36)	675 (39)	603 (39)
Stage 2	33,288 (20)	5,080 (18)	588 (20)	445 (21)	345 (20)	309 (20)
Stage 3	48,738 (29)	8453 (30)	766 (26)	589 (27)	456 (26)	392 (25)
Stage 4	5,497 (3)	1006 (4)	130 (4)	94 (4)	69 (4)	63 (4)
Unknown	15,825 (10)	2,882 (10)	349 (12)	252 (12)	206 (12)	181 (12)
**Province**						
Eastern	29,701 (18)	3,523 (13)	553 (19)	464 (21)	373 (21)	360 (23)
Lusaka	86,688 (52)	17,754 (63)	1,284 (44)	884 (41)	750 (43)	626 (40)
Southern	24,864 (15)	2,714 (10)	507 (18)	384 (18)	291 (17)	245 (16)
Western	24,211 (15)	4,120 (15)	548 (19)	431 (20)	337 (19)	317 (20)
**Year of enrollment**						
2004–2006	16,198 (10)	1,723 (6)	142 (5)	93 (4)	80 (5)	68 (4)
2007–2009	41,050 (25)	5,538 (20)	570 (20)	435 (20)	354 (20)	329 (21)
2010–2012	53,594 (32)	9,148 (33)	1,015 (35)	758 (35)	639 (36)	573 (37)
2013–2015	54,622 (33)	11,702 (42)	1,165 (40)	877 (41)	678 (39)	578 (37)
**Year of ART initiation**						
2004–2006	15,330 (9)	1,607 (6)	164 (6)	126 (6)	101 (6)	92 (6)
2007–2009	34,144 (21)	4,497 (16)	442 (15)	323 (15)	263 (15)	239 (15)
2010–2012	48,288 (29)	7,726 (27)	854 (30)	652 (30)	557 (32)	502 (32)
2013–2015	67,702 (41)	14,281 (51)	1,432 (50)	1,062 (49)	830 (47)	715 (46)
**Duration of ART (days)**	1,142 (390–2,139)	535 (98–1,492)	592 (104–1,496)	611 (119–1,508)	673 (159–1,535)	721 (174–1,561)
**Disclosure of HIV status to family or friend**						
No	2,580 (2)	642 (2)	63 (2)	41 (2)	30 (2)	21 (1)
Yes	142,021 (86)	24,027 (85)	2,472 (85)	1,844 (85)	1,489 (85)	1,316 (85)
Unknown	20,863 (13)	3,442 (12)	357 (12)	278 (13)	232 (13)	211 (14)
**Education level**^c^						
None	9,660 (6)	1,674 (6)	226 (8)	170 (8)	124 (7)	115 (7)
Lower	48,175 (29)	7,606 (27)	813 (28)	599 (28)	467 (27)	426 (28)
Upper	62,154 (38)	11,542 (41)	1,098 (38)	833 (39)	687 (39)	583 (38)
College	6,398 (4)	1,107 (4)	113 (4)	98 (5)	86 (5)	73 (5)
Unknown	39,077 (24)	6,182 (22)	642 (22)	463 (21)	387 (22)	351 (23)
**Marital status**						
Single	14,965 (9)	3,130 (11)	320 (11)	239 (11)	198 (11)	177 (11)
Married	86,091 (52)	14,422 (51)	1,493 (52)	1,147 (53)	965 (55)	859 (56)
Divorced	16,958 (10)	3,103 (11)	342 (12)	250 (12)	180 (10)	160 (10)
Widowed	16,125 (10)	2,211 (8)	228 (8)	174 (8)	126 (7)	110 (7)
Unknown	31,325 (19)	5,245 (19)	509 (18)	353 (16)	282 (16)	242 (16)
**Facility**						
Rural health center	16,547 (10)	3,163 (11)	633 (22)	536 (25)	434 (25)	403 (26)
Urban health center	92,216 (56)	17,667 (63)	1,476 (51)	1,047 (48)	874 (50)	779 (50)
Hospital	56,701 (34)	7281 (26)	783 (27)	580 (27)	443 (25)	366 (24)

Values are *N* (%) or median (IQR).

^a^Missing for 33,296 (20.1%).

^b^Missing for 22,374 (13.5%).

^c^Lower = lower/mid-basic schooling; upper = upper-basic/secondary school; college = college/university.

**Fig 2:**
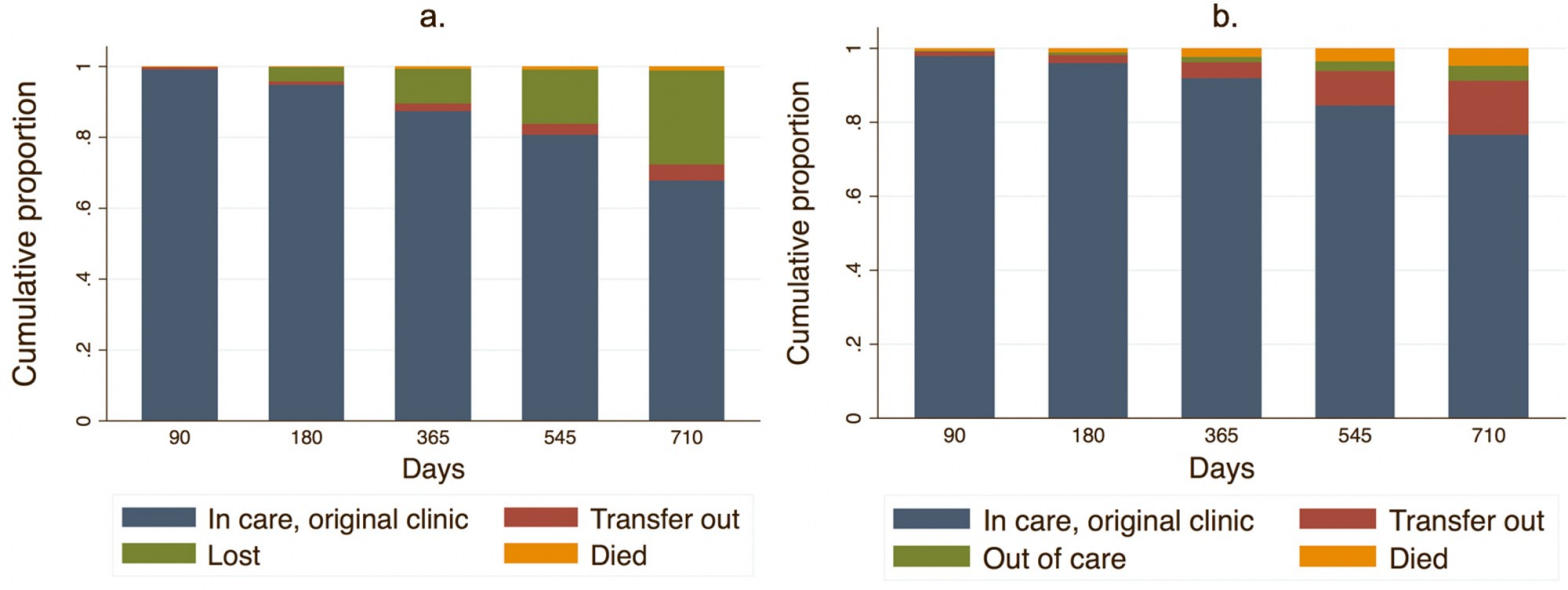
Total ART cohort (*N* = 165,464): Estimated naïve and revised cumulative proportion of patients in care over time. Naïve (a) and revised (b) estimates. *X* axis represents days on ART during the cohort observation period (1 August 2013–31 July 2015). “Transfer out” includes official transfers and—for revised estimates—unofficial transfers to a new clinic ascertained by patient self-report.

Among all patients at 2 years, using only EMR data, we found that 67.7% of patients were retained at the original clinic (95% CI 67.3%–68.3%), 26.5% were lost (95% CI 26.1%–26.8%), 4.6% had officially transferred to a new facility (95% CI 4.5%–4.7%), and 1.2% had died (95% CI 1.1%–1.2%) (Fig 2; S3 Table)—indicating that 72.3% (95% CI 71.8%–73.0%) were retained at 2 years (at original clinic or transferred). After incorporating updated tracing outcomes, the revised 2-year estimates suggested that 76.5% (95% CI 76.0%–76.9%) were retained at the original clinic, 14.7% (95% CI 14.5%–14.9%) had officially or unofficially transferred to a new site, 3.9% (95% CI 3.8%–4.1%) were alive and out of care, and 4.9% (95% CI 4.8%–5.0%) had died (Fig 2; S3 Table), resulting in an updated estimate of 91.2% (95% CI 90.5%–91.8%) retained (at original clinic or transferred) at 2 years.

**Fig 3:**
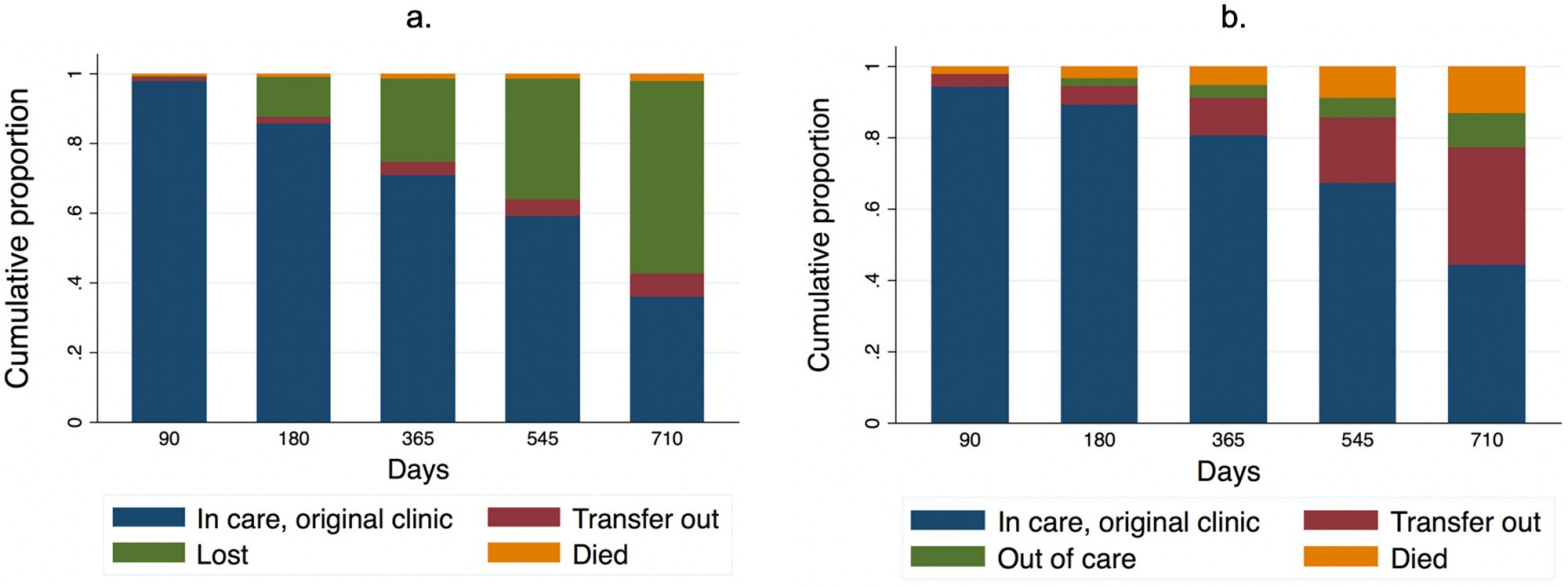
New ART initiators (*N* = 49,129): Estimated naïve and revised cumulative proportion of patient in care over time. Naïve (a) and revised (b) estimates. *X* axis represents days since ART initiation (all initiated on or after 1 August 2013). “Transfer out” includes official transfers and unofficial transfers to a new clinic.

Compared to the total ART cohort, new ART initiators were less likely to be retained. Two-year estimates for this group using only EMR data showed 35.9% were retained (95% CI 31.8%–39.9%), 55.1% were lost, 6.8% transferred (95% CI 6.2%–7.5%), and 2.2% died (95% CI 1.7%–2.8%) (Fig 3; S4 Table), with a total of 42.7% (95% CI 38.1%–47.0%) retained in care (at original clinic or transferred). Revised Aalen–Johansen estimates incorporating tracing outcomes through probability weights showed a cumulative proportion of 44.2% who were retained in care at original clinic (95% CI 40%–48%), 33.1% who had transferred to new clinics (95% CI 30.7%–35.6%), 9.6% who were out of care (95% CI 8.7%–10.5%), and 13.1% who had died (95% CI 12.2%–14.1%) (Fig 3; S4 Table), resulting in updated estimates of 77.3% (95% CI 70.5%–84.0%) retained (at the original clinic or transferred) at 2 years.

**Fig 4:**
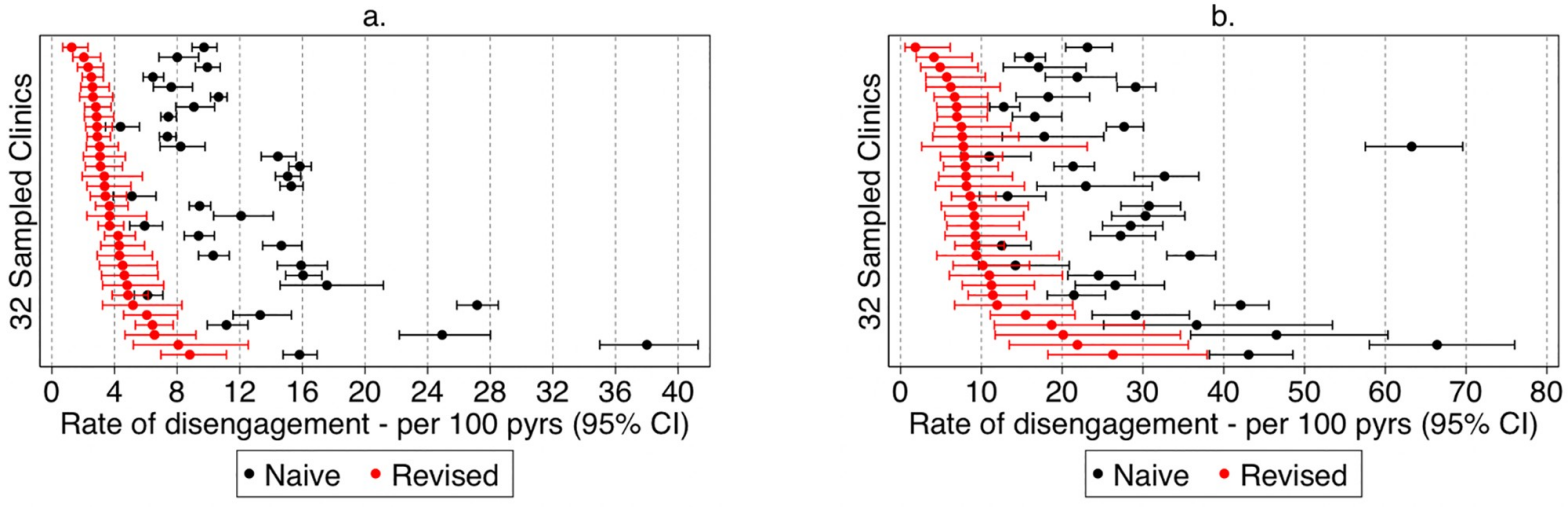
Naïve and revised facility-level rates of disengagement at the 32 sampled clinics among all ART users and new ART initiates. (a) All ART users; (b) new ART initiates. pyrs, person-years.

Revised rates of stopping care varied markedly across health facilities, ranging between 1.3 and 8.8 per 100 person-years (pyrs) in the total cohort (Fig 4a), and 1.8 and 26.3 per 100 pyrs among the new ART initiators (Fig 4b), and across the 4 provinces, ranging from 4.0 (95% CI 3.5–4.5) per 100 pyrs in Eastern Province to 5.5 (95% CI 4.9–6.2) per 100 pyrs in Lusaka Province in the total ART cohort, and from 9.1 (95% CI 7.5–11.0) per 100 pyrs in Eastern Province to 12.5 (95% CI 10.3–15.2) per 100 pyrs in Lusaka Province among new ART initiates (S3 Fig).

At the individual level, the characteristics most strongly associated with disengagement were male sex (hazard ratio [HR] 1.82; 95% CI 1.47–2.25; *p <* 0.001) and years on ART (HR 0.81; 95% CI 0.79–0.84; *p <* 0.001). In addition, a low CD4 count or being divorced as compared to married at enrollment showed an association with higher disengagement (Table 2). In the sample of new ART initiates, enrollment CD4 count, WHO stage, and divorce were associated with disengagement.

**Table 2:** Naïve and revised multivariable analyses of factors associated with disengagement (died or alive and out of care).

Baseline characteristic	Total ART cohort (*N* = 165,464)						New ART initiators (*N* = 49,129)					
	Naïve^a^			Revised^a^^,^^b^			Naïve^a^			Revised^a^^,^^b^		
	HR	95% CI	*p*-Value	HR	95% CI	*p*-Value	HR	95% CI	*p*-Value	HR	95% CI	*p*-Value
**Sex**			<0.001			<0.001			<0.001			0.006
Female	1			1			1			1		
Male	1.36	1.28–1.45		1.82	1.47–2.25		1.25	1.16–1.34		1.50	1.13–1.99	
**Age (per 10 years)**	0.73	0.71–0.76	<0.001	0.86	0.71–1.02	0.095	0.76	0.73–0.79	<0.001	0.90	0.73–1.11	0.311
**Enrollment CD4 count (per 50 cells/μl)**			<0.001			0.011			<0.001			<0.001
0–100	1			1			1			1		
101–200	0.91	0.85–0.96		1.13	0.83–1.59		0.83	0.74–0.92		0.84	0.46–1.53	
201–350	0.82	0.77–0.86		0.82	0.61–1.10		0.73	0.68–0.80		0.70	0.45–1.09	
351–500	0.81	0.75–0.87		0.57	0.37–0.88		0.73	0.67–0.81		0.33	0.17–0.64	
≥501	0.87	0.81–0.95		0.68	0.44–1.09		0.76	0.67–0.85		0.53	0.28–1.02	
**WHO stage**			<0.001			0.062			<0.001			0.005
1	1			1			1			1		
2	1.06	0.98–1.14		1.27	0.92–1.72		1.08	0.95–1.22		1.64	1.07–2.52	
3	1.27	1.19–1.35		1.13	0.83–1.52		1.39	1.24–1.56		1.13	0.66–1.94	
4	1.53	1.37–1.71		1.83	1.05–3.18		1.86	1.45–2.37		2.04	1.01–4.12	
**Time on ART prior to study enrollment (per year)**	0.81	0.79–0.84	<0.001	0.77	0.68–0.86	<0.001	—	—	—	—	—	—
**Province**			<0.001			0.515			<0.001			0.581
Lusaka	1			1			1			1		
Eastern	0.70	0.66–0.75		1.07	0.75–1.53		0.64	0.47–0.87		0.99	0.51–1.93	
Southern	0.51	0.36–0.73		0.73	0.44–1.22		0.48	0.35–0.66		0.65	0.35–1.21	
Western	0.96	0.57–1.62		1.05	0.53–2.08		0.87	0.55–1.37		0.73	0.33–1.61	
**Original facility type**			0.382			0.051			0.661			0.629
Urban health center	1			1			1			1		
Rural health center	1.06	0.68–1.64		0.77	0.47–1.24		0.97	0.63–1.50		1.13	0.52–2.52	
Hospital	0.81	0.56–1.16		0.65	0.46–0.92		0.85	0.60–1.21		0.82	0.49–1.37	
**Facility size (per 1,000 patients)**	0.95	0.90–1.00	0.051	0.93	0.85–1.02	0.123	0.95	0.91–0.99	0.016	0.93	0.83–1.04	0.194
**Marital status**			<0.001			0.007			0.007			0.040
Married	1			1			1			1		
Single	1.12	1.03–1.22		1.23	0.79–1.91		1.05	0.94–1.18		1.46	0.98–2.19	
Divorced	1.17	1.10–1.25		1.77	1.29–2.45		1.14	1.06–1.24		1.65	1.06–2.57	
Widowed	1.12	1.06–1.19		1.12	0.79–1.58		1.08	0.96–1.21		1.58	0.89–2.81	
**Disclosed HIV status to family or friend**			0.086			0.751			0.006			0.463
No	1			1			1			1		
Yes	0.88	0.77–1.02		1.12	0.5–2.23		0.78	0.66–0.93		0.74	0.34–1.64	
**Education level**^c^			0.069			0.453			0.008			0.621
None	1			1			1			1		
Lower	0.85	0.75–0.97		1.02	0.70–1.50		0.85	0.76–0.97		1.19	0.71–2.00	
Upper	0.90	0.79–1.02		1.15	0.79–1.68		0.90	0.78–1.03		1.35	0.71–2.56	
College	0.93	0.80–1.07		1.53	0.80–2.93		0.83	0.68–1.00		1.84	0.75–4.52	

^a^Inverse probability weighting used to account for <20% missing values for education, disclosure, marital status, WHO stage, and CD4 count.

^b^Inverse probability sampling weights further applied to generate revised estimates; robust standard errors for clustering at the facility level.

^c^Lower = lower/mid-basic schooling; upper = upper-basic/secondary school; college = college/university.

HR, hazard ratio.

Among the 86,688 patients who initiated ART during the study period in Lusaka Province (where we sought to estimate the prevalence of viremia), 68,934 were retained in care and 17,754 were lost to follow-up. Of a random sample of 798 lost patients who were eligible for tracing, 400 (50.1%) could not be traced, and for 255 (32.0%), samples could not be obtained due to either refusals or logistical challenges, resulting in 143 (17.9%) DBS viral load samples. Characteristics were similar for eligible patients with and without viral load samples (S5 Table). In a systematic sample of retained patients, we obtained 901 DBS viral load samples. In combination, we analyzed 1,044 DBS viral load results (S1 Fig). After applying inverse probability weights for sampling and nonresponse, and bias correction for known misclassification of DBS-based HIV RNA levels (as compared to plasma HIV RNA levels), we found the prevalence of viremia among patients retained at their original health facility (using a threshold of 1,000 copies/ml) to be 18.1% (95% CI 14.0%–22.3%). Among the lost patients, which included both those reporting no care as well as those who unofficially transferred, 71.3% (95% CI 58.2%–84.4%) were viremic. Unofficial transfers and patients out of care had a prevalence of viremia of 49.8% (95% CI 28.1%–71.4%) and 83.9% (95% CI 67.2%–98.8%), respectively. Incorporating results among those lost and traced into the underlying cohort using probability weights yielded an overall prevalence of viremia of 24.7% (95% CI 21.0%–29.3%). In multivariable regression using pre-therapy patient characteristics (Table 3), male sex, younger age, and lower ART initiation CD4 count were associated with viremia. In a model with current care status, this factor was most strongly associated with viremia. Male sex and time on ART diminished in significance, but younger age remained strongly associated with viremia in the model with current care status.

**Table 3:** Factors associated with viremia (defined as ≥1,000 copies/ml) after application of sampling weights and bias correction for sensitivity and specificity of dried blood spot viral load measurements (*N* = 1,044).

Predictor	No adjustment for current care status			Adjusted for current care status		
	RR	95% CI	*p*-Value	RR	95% CI	*p*-Value
**Sex**			0.029			0.188
Female	Ref			Ref		
Male	1.75	1.06–2.88		1.46	0.83–2.56	
**Age (per 10 years)**	0.58	0.42–0.79	0.001	0.62	0.44–0.89	0.009
**ART initiation CD4 count (per 50 cells/μl)**	0.87	0.79–0.96	0.006	0.87	0.78–0.97	0.011
**Time on ART (per year)**	0.91	0.83–1.01	0.065	0.98	0.87–1.09	0.675
**Facility size (per 1,000 patients)**	1.04	0.95–1.13	0.373	1.09	0.99–1.20	0.095
**Care status**						<0.001
In care, original clinic	—	—	—	1		
Unofficial transfer	—	—	—	3.29	1.13–9.54	
Out of care	—	—	—	17.44	5.58–54.47	

RR, risk ratio.

## Discussion

We combined targeted supplemental data collection (for updated care status and viral loads) with large-scale data from a national EMR system to advance our understanding of the public health response to HIV in Zambia. First, we found that even though a large percentage of patients missed visits and became lost to follow-up, most patients returned to care and relatively few patients stopped care altogether. Second, these lapses in retention varied markedly from 1.8 to 26.3 per 100 pyrs among new ART initiates attending 32 health facilities across 4 provinces we studied, differences that were incompletely explained by measured patient and facility characteristics. Third, unsuppressed HIV RNA levels in a population of treated patients rose substantially when lost-to-follow-up patients were included in estimates. In this study, viremia rose by 7% on an absolute scale and nearly 40% on a ratio scale when lost patients were included in the estimates. Patients who were retained within the greater health system, but not at their original clinics, contributed substantially to the total viremia in the population: Patient-initiated transfers of care were not well coordinated and safe. These findings suggest that public health HIV treatment services in Zambia, while accomplishing an enormous task and saving thousands of lives, are of uneven success: Many patients do not achieve optimal sustained engagement, and they experience viremia and therefore attenuated clinical benefits of HIV treatment.

When compared to the large-scale cross-sectional ZAMPHIA—which documents a prevalence of viral suppression among current HIV ART users of 90% in Zambia [8]—our longitudinal data suggest several additional observations. First, we find the prevalence of viremia in a population treated within the last 2 years to be 25% when those lost to follow-up are incorporated into estimates. This is substantially higher than the 10% estimated in ZAMPHIA. Although this difference could be due in part to measurement error (i.e., limited sensitivity and specificity) of DBSs, we sought to manage these consequences through bias correction methods. Another important possibility, however, is that cross-sectional studies do not fully capture those patients who had been on treatment, but who stopped treatment prior to their participation in the survey. If these patients do not admit to previous treatment (due to social desirability bias) or if instruments only ask about current HIV treatment, the denominator could be artificially small (and viral suppression overestimated) compared to this analysis. In either case, we found our internal estimates of viral suppression among those in clinic care to be much higher when lost-to-follow-up patients were included.

The revised estimates of retention and viral suppression do not just change the numerical estimates, but further illustrate that retention is a multidimensional, complex outcome that likely requires adaptive, innovative, longitudinal public health practices to improve. On the one hand, the prevalence of true disengagement (i.e., being alive and out of care) is much lower in reality than as shown by estimates using EMR data alone. On the other hand, patients who drop out of care at one site and reenter at another are much more likely to be viremic (at approximately 50%). Collectively, these 2 observations direct our attention to an important reality in public health chronic disease management: Patients may move their residence, and their prioritization of treatment may wax and wane, and these changes represent periods of vulnerability [20–22]. Innovations to improve the effectiveness of ART programs, such as differentiated service delivery, as well as others, must adapt to patients’ lived realities in order to support durable, long-term engagement in care and viral suppression [23–25].

Ongoing efforts to enhance retention and viral suppression are underway, both in the environment of this study in Zambia and beyond, but will need continued monitoring, including among those inevitably lost to follow-up. Zambia is rapidly scaling up targeted quality improvement activities in response to these findings, including wider use of differentiated service delivery models. Although the improvement of estimates of retention through tracing a sample of lost patients has been demonstrated in sub-Saharan Africa [5,26,27], this analysis also highlights the marked heterogeneity across facility-level estimates. The heterogeneity implies that the intensification or prioritization of retention strategies should be targeted, to be most efficient, and one should endeavor to understand facility-level and facility–patient interaction dynamics when implementing support efforts. This targeting of health system improvements is aligned with current strategic thinking about targeting resources to those most in need [5,25,26]. In addition, interventions that facilitate reengagement in care for those who are found to be disengaged from care are needed. Data suggest that early tracing after a missed visit (within 1 week) can improve patient contact and return to care [27–29], an intervention that should be considered in this setting, but must occur alongside improvements in EMR systems and data management to minimize misclassification and wasted tracing efforts.

In our analysis, we observed that the strongest predictors of disengagement were male sex and advanced HIV disease [5,27]. The recent introduction of the concept of differentiated care for patients with advanced HIV disease could, over time, have an impact on this higher risk of disengagement among those who present late to HIV services [24]. Effective approaches to retaining men in care in sub-Saharan Africa are less clearly defined [30,31]; however, interventions such as home, mobile, or workplace ART distribution and financial incentives that target men should be conceptualized with consideration of the unique facility characteristics and community dynamics relevant to the settings where these services may be implemented. Conceptually, those lost to follow-up will have greater prevalence of viremia than those in care. If this fraction of lost patients is large, then their overall contribution to viremia in a population is important. These data offer proof of that concept. Among those in care, only 1 in 6 patients are viremic, whereas over half of those lost to follow-up are viremic. Of note, patients who silently transferred (i.e., had no official transfer documentation) between facilities also had a high risk of viremia, highlighting the contribution of treatment gaps during transfer to the overall infectiousness of this community. Efforts should be made to ensure rapid reengagement in care among those with missed visits by simplifying transfer systems and educating patients and staff to monitor and document reengagement processes at original or new facilities.

This study has a number of limitations. Our sample, even though randomly selected, was affected by imperfect response rates: We did not ascertain outcomes in all those who were traced. In addition, we ascertained true care status among lost patients by self-report, which could be influenced by social desirability bias. Self-reported retention status, however, was highly associated with viral load, lending credibility to this measurement. Furthermore, our competing risk estimates assumed that unofficial and official transfers remained engaged in care, an assumption that could lead to an overestimation of retention. Viral load measurements used DBSs, which have known limitations in sensitivity and specificity compared to the gold standard plasma-based assay; we however used established methods of bias analysis to correct for DBS inaccuracy.

This study demonstrates how a strategy of sampling and tracing of lost patients can be used to generate revised estimates of retention and viremia at a population level. For Zambia these estimates reflected better overall retention than routinely collected program data but also highlighted significant gaps in care, and marked variation of overall retention at the facility level among those retained, contributing to high overall viremia in the ART cohort. Substantial efforts need to be made to tailor services to the needs of patients in order to reduce lapses in care and maintain long-term viral suppression. Furthermore, understanding facility- and community-based barriers to retention in care and addressing these barriers remain critical to attaining the UNAIDS 90-90-90 targets.

## Supporting information

S1 Appendix Protocol. (PDF)

S2 Appendix STROBE checklist. (DOCX)

S3 Appendix Survey tool. (DOCX)

S4 Appendix Viral load bias misclassification technique. (PDF)

S1 Fig Viral load sampling and weighting flow diagram. (TIF)

S2 Fig ART cohort sampling weight flow diagram. (TIF)

S3 Fig Provincial mortality rates. (TIFF)

S1 Table Test of proportional hazards assumption. (DOCX)

S2 Table Characteristics of new ART initiates. (DOCX)

S3 Table Naïve and revised retention estimates: Total ART cohort. (DOCX)

S4 Table Naïve and revised retention estimates: New ART initiates. (DOCX)

S5 Table Characteristics of patients eligible for viral load sampling. (DOCX)

## Abbreviations

CIDRZ: Centre for Infectious Disease Research in Zambia
DBS: dried blood spot
EMR: electronic medical record; pyrs, person-years
ZAMPHIA: Zambia Population-based HIV Impact Assessment
