# Correction: Retention and viral suppression in a cohort of HIV patients on antiretroviral therapy in Zambia: Regionally representative estimates using a multistage-sampling-based approach

**DOI:** 10.1371/journal.pmed.1002918

**Published:** 2019-08-30

**Authors:** Izukanji Sikazwe, Ingrid Eshun-Wilson, Kombatende Sikombe, Nancy Czaicki, Paul Somwe, Aaloke Mody, Sandra Simbeza, David V. Glidden, Elizabeth Chizema, Lloyd B. Mulenga, Nancy Padian, Chris J. Duncombe, Carolyn Bolton-Moore, Laura K. Beres, Charles B. Holmes, Elvin Geng

## Notice of Republication

This article was republished on 7^th^ August, 2019, to remove S1 Data file which is part of a larger data set which requires additional permission for access. Please download this article again to view the correct version. To request data access, contact the CIDRZ Ethics and Compliance Committee Chair/Chief Scientific Officer, Dr. Roma Chilengi, Roma.chilengi@cidrz.org), or the Secretary to the Committee/Head of Research Operations, Ms. Hope Mwanyungwi, Hope.Mwanyungwi@cidrz.org), mentioning the intended use for the data.
