# Supplementary material for: Retention and viral suppression in a cohort of HIV patients on antiretroviral therapy in Zambia: Regionally representative estimates using a multistage-sampling-based approach
Source: PLoS Med. 2019 May 31;16(5):e1002811. doi: 10.1371/journal.pmed.1002811 (PMC6544202; doi:10.1371/journal.pmed.1002811)
Supplement: S2 Appendix — (DOCX) [file pmed.1002811.s002.docx]

|  | **Item No** | **Recommendation** | **Response:** |
| --- | --- | --- | --- |
| **Title and abstract** | | | |
|  | 1 | a) Indicate the study’s design with a commonly used term in the title or the abstract | The design is included in the title - "sampling based approach". This is further explained in the abstract method section "we used a multi-staged sampling approach to select facilities and random sampling to identify a representative sample of LTFU…" |
|  |  | b) Provide in the abstract an informative and balanced summary of what was done and what was found | This has been done |
| **Introduction** | | | |
| Background | 2 | Explain the scientific background and rationale for the investigation being reported | To assess the overall effectiveness of HIV care and treatment cascade in 4 provinces in Zambia by extending the epidemiologic application of a sampling-based approach to generate revised regional estimates of retention and in one province HIV RNA suppression. In HIV prevalent settings with large absolute numbers of patients, tracing all lost to follow-up patients to comprehensively account for patient outcomes is not a viable strategy. We instead identified a numerically small but randomly selected sample of lost patients and incorporated their outcomes into underlying facility patient populations. ***See introduction section paragraph 2*** |
| Objectives | 3 | State specific objectives, including any prespecified hypotheses | To determine the overall patient retention in 4 provinces in Zambia using a sampling-based approach to generate revised regional estimates of retention and in one province HIV RNA suppression in patients retained in care and those LTFU. ***See introduction section paragraph 3*** |
| **Methods** | | | |
| Study design | 4 | Present key elements of study design early in the paper | Multi-staged sampling of health facilities and LTFU participants. ***See Methods Section - Patients and sampling*** |
| Setting | 5 | Describe the setting, locations, and relevant dates, including periods of recruitment, exposure, follow-up and data collection | Study conducted in 32 health facilities across 4 provinces (Lusaka, Western, Eastern and Southern) in Zambia. Participants who sought care between August 2013 and July 2015 and were LTFU after these dates were sampled and tracked between October 2015 to June 2016. ***See Methods section - Patients and Sampling*** |
| Participants | 6 | a) Give the eligibility criteria, and the sources and methods of selection of participants. Describe methods of follow-up | HIV+ adults 18 years and older, who sought care between August 2013 - July 2015, in 4 provinces in Zambia, where CIDRZ supports MOH HIV services. Patients flagged as LTFU or late by the electronic medical record system were generated and from this list, and a random sample of 10% of them were selected for tracing to ascertain their vital status. Participants were followed up via phone calls or in person. ***See Methods section under patients and sampling*** |
|  |  | b) For matched studies, give matching criteria and number of exposed and unexposed | Not applicable |
| Variables | 7 | Clearly define all outcomes, exposures, predictors, potential confounders, and effect modifiers. Give diagnostic criteria if applicable | We define all outcomes in detail : In the analysis of data obtained from the EMR (and without data from tracing) we estimated the prevalence of four care states over time using the Aalen Johansen method (13): (1) alive and in care at original clinic, (2) transferred to a new facility (i.e., an official transfer), (3) lost to follow-up or (4) died. We then estimated revised care states: (1) alive and in care at the original clinic, (2) transfer to a new clinic (i.e., including both official and silent transfers), (3) alive but out of care (more than 90 days late for last appointment at time of sampling), or (4) died: ***Methods section - Analyses paragraph 2.*** We present all counfounders/effect modifiers in ***Table 1.*** |
| Data sources/ measurements | 8 | For each variable of interest, give sources of data and details of methods of assessment. Describe comparability of assessment methods if there is more than one group | The electronic medical record and study tools. ***See Methods section, procedures and measurements*** |
| Bias | 9 | Describe any efforts to address potential sources of bias | Poor response rates were a concern in this analysis. For all analyses non-respondents were treated as missing completely at random. We did not conduct alternate sensitivity analyses to explore this and highlight this potential source of bias in our limitations. ***See Discussion – paragraph 6*** |
| Study size | 10 | Explain how the study size was arrived at | We have detailed in the manuscript our sampling strategy for the entire cohort and those who were included in the viral load analysis – this is detailed in: Methods section, Patients and Sampling, Fig 1, and S1 Fig. |
| Quantitative variables | 11 | Explain how quantitative variables were handled in the analyses. If applicable, describe which groupings were chosen and why | All quantitative variables were included in models as continuous, except CD4 count which were categorized into recognized CD4 thresholds representing levels of immunosuppression. |
| Statistical methods | 12 | (a ) Describe all statistical methods, including those used to control for confounding | A detailed description of all statistical methods, weighting strategies and viral load bias misclassification techniques are presented in the methods section and supplementary materials. Cox proportion hazards models were used to identify baseline characteristics associated with disengagement, inverse probability sampling weights were applied to generate revised estimates. ***See Methods sections: Analyses*** |
|  |  | (b ) Describe any methods used to examine subgroups and interactions | We present findings for both the total ART cohort and the sub-group of new ART initiates. No interactions were explored |
|  |  | (c) Explain how missing data were addressed | Inverse probability weights were used to account for missing data, this is described in the manuscript - ***See Methods section: Analyses*** |
|  |  | (d ) If applicable, explain how loss to follow-up was addressed | Not applicable, as we traced lost patients |
|  |  | (e ) Describe any sensitivity analyses | No sensitivity analyses were conducted |
| **Results** |  |  |  |
| Participants | 13 | (a) Report numbers of individuals at each stage of study—eg numbers potentially eligible examined for eligibility, confirmed eligible, included in the study, completing follow-up, and analysed | This is included in the consort - fig 1. ***See Results section, paragraph 1 and figure 1*** |
|  |  | (b) Give reasons for non-participation at each stage | Section Results section, figure 1 |
|  |  | (c) Consider use of a flow diagram | Done, as above |
| Descriptive Data | 14 | (a) Give characteristics of study participants (eg demographic, clinical, social) and information on exposures and potential confounders | This is done. ***See Results section, paragraph 2, table 1*** |
|  |  | (b) Indicate number of participants with missing data for each variable of interest | This is done. ***See Results section, paragraph 1 and 2, table 1*** |
|  |  | (c) Summarise follow-up time (eg, average and total amount) | All efforts to track lost patients were done between Oct 2015 and June 2016 as described in the methods section |
| Outcome data | 15 | Report numbers of outcome events or summary measures over time | The updated tracing outcomes of the cumulative estimates of patients in each state over time for the total ART cohort and the new ART cohort have been reported in the Results section, paragraph 3 and in Fig 2, Fig 3 and S3 & S4 Tables. |
| Main Results | 16 | (a) Give unadjusted estimates and, if applicable, confounder-adjusted estimates and their precision (eg, 95% confidence interval). Make clear which confounders were adjusted for and why they were included | This has been done as stated above in #15. We provide naïve and revised estimates based on tracing outcomes. Models however only present multivariable adjusted models |
|  |  | (b) Report category boundaries when continuous variables were categorized | CD4 categories are presented in Table 1 and throughout |
|  |  | (c ) If relevant, consider translating estimates of relative risk into absolute risk for a meaningful time period | We present primarily hazard ratios and cumulative proportion for our results. For the viraemia analysis we do present relative risk after adjustment for confounders, we also present the prevalence of viraemia across care states and feel that these results sufficiently present the findings of interest for this outcome. |
| Other analyses | 17 | Report other analyses done—eg analyses of subgroups and interactions, and sensitivity analyses | We present all analyses in the paper with additional analysis methods in the supplementary materials. There were no additional analyses not presented in the full manuscript. |
| **Discussion** |  |  |  |
| Key Results | 18 | Summarise key results with reference to study objectives | In our introduction we highlight that “Assessments of retention on treatment and HIV RNA suppression levels after HIV treatment initiation in routine program settings represent the backbone of data-driven public health efforts to bring the epidemic under control in high prevalence settings such as Zambia.” Our results and discussion reflect on this and highlight that we provide better estimates of retention and viraemia than routine program monitoring data |
|  | 19 | Discuss limitations of the study, taking into account sources of potential bias or imprecision. Discuss both direction and magnitude of any potential bias | Yes, we present limitations and their likely impact on our study findings in the limitations section: Discussion, paragraph 6 |
| Interpretation | 20 | Give a cautious overall interpretation of results considering objectives, limitations, multiplicity of analyses, results from similar studies, and other relevant evidence | We have been careful to point out that these are ‘estimates’ and that there are limitations in our analysis and findings. |
| Generalisability | 21 | Discuss the generalisability (external validity) of the study results | The study methodology and results can be applied to the remaining provinces in Zambia and the region which has seen a rapid growth with varying loss of patients across the treatment cascade. See Discussion section, paragraph 7 |
| **Other information** |  |  |  |
| Funding | 22 | Give the source of funding and the role of the funders for the present study and, if applicable, for the original study on which the present article is based | Funding was provided by the Bill and Melinda Gates Foundation, through grant number OPP1105071. See section on Funding below authors information |
