# Supplementary material for: Retention and viral suppression in a cohort of HIV patients on antiretroviral therapy in Zambia: Regionally representative estimates using a multistage-sampling-based approach
Source: PLoS Med. 2019 May 31;16(5):e1002811. doi: 10.1371/journal.pmed.1002811 (PMC6544202; doi:10.1371/journal.pmed.1002811)
Supplement: S4 Appendix — (PDF) [file pmed.1002811.s004.pdf]

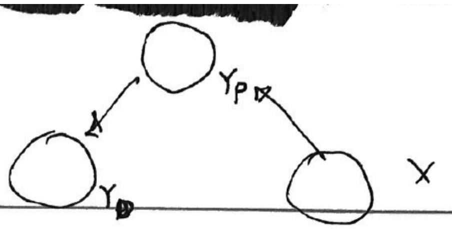

Y_P : RNA plasma , Y_D : RNA DBS, X : covs
1 = detected, 0 = not

sens : $P(Y_D = 1 \mid Y_P = 1, X) = \gamma_1$ non-differential ~~cind~~ indep of X, not edge X and Y_D

spec : $P(Y_D = 0 \mid Y_P = 0, X) = 1 - \gamma_0$ $\gamma_0 = P(Y_D = 1 \mid Y_P, X)$ non-differential

Our model : logistic or RR model for X and Y_P

Y_P not observed but misclassification probs "known"
for generalized linear model theory and Neuhaus (1999)

$\mu_P(X) = P(Y_P = 1 \mid X)$ ~~follows a GLM~~

$g_P\{\mu_P(X)\} = \eta_P = X\beta_P$ $Y_P \sim$ binomial
g_P = logit, logistics reg ; g_P = log, RR reg

Neuhaus '99 Note : $\mu(X) = P(Y_D = 1 \mid X) = \gamma_0 + (\gamma_1 - \gamma_0)\mu_P(X)$
which is a GLM where link g is

$$g\{\mu(X)\} = g_P\left\{\frac{\mu_P(X) - \gamma_0}{(\gamma_1 - \gamma_0)}\right\} = \eta_P$$

$$\mu(X) = g_P^{-1}\{\eta_P\}(\gamma_1 - \gamma_0) + \gamma_0$$

g_P : logit for logistic g_P : log for RR

This is a GLM with a modified link function

Stata allows you to write these. I wrote one for ORs and one for RRs.

Need to calculate

	logit	log
$\frac{\partial \mu}{\partial \eta}$	$(\gamma_1-\gamma_0)\,\text{expit}(\eta)(1-\text{expit}(\eta))$	$(\gamma_1-\gamma_0)\,e^{\eta}$
$\frac{\partial^2 \mu}{\partial \eta^2}$	$(\gamma_1-\gamma_0)\frac{e^{\eta}(1-e^{\eta})}{(1+e^{\eta})^3}$	$(\gamma_1-\gamma_0)\,e^{\eta}$

These are programmed in two ado file "mislogit" and "mislog"

From Ingrid: $\gamma_1 = 0.808$ (sens), $1-\gamma_0 = 0.873$ (spec)

$\Rightarrow \gamma_0 = 0.127$

$\gamma_1 - \gamma_0 = 0.681$

type "sysdir" in Stata. It tells you where "ado" files go. For me its

~/Library/Application support/stata/ado/personal

If you put the ado files there, the code works

```
program mislog
version 14.2
args todo eta mu return
if `todo' == -1 {
            /* Set global macros for output */
            global SGLM_lt "misclassified log"
            global SGLM_lf "log( (p-0.127)/0.681)"
            exit
}
if `todo' == 0 {
            /* set eta =g(mu) */
            generate double `eta' = log( ((`mu' - 0.127)/0.681) )
            exit
}
if `todo' == 1 {
            /* set g^{-1}(eta) */
            generate double `mu' = exp(`eta')*0.681 + 0.127
            exit
}
if `todo' == 2 {
            /* set return = dmu/deta */
   local d2 = 0.681*exp(`eta')
            generate double `return' = 0.681*exp(`eta')
exit
}
if `todo' == 3 {
            /* set return =d2mu/dmu2  */
            generate double `return' = 0.681*exp(`eta')
            exit
}
display as error "Unknown call to glm link function"
exit 198
end
```

```
program mislogit
version 14.2
args todo eta mu return
if `todo' == -1 {
            /* Set global macros for output */
            global SGLM_lt "logit with missclassification"
            global SGLM_lf "logit(((p - 0.127)/0.681))"
            exit
```

```
}
if `todo' == 0 {
	/* set eta =g(mu) */
	generate double `eta' = logit( ((`mu' - 0.127)/0.681) )
	exit
}
if `todo' == 1 {
	/* set g^{-1}(eta) */
	generate double `mu' = (invlogit(`eta')*0.681) + 0.127
	exit
}
if `todo' == 2 {
	/* set return = dmu/deta */
	generate double `return' = 0.681*invlogit(`eta')*(1-invlogit(`eta'))
exit
}
if `todo' == 3 {
	/* set return =d2mu/dmu2  */
	generate double `return' = 0.681*( exp(`eta')*(1-exp(`eta')) )/( (1+exp(`eta'))^3 )
	exit
}
display as error "Unknown call to glm link function"
exit 198
end
```
