# Supplementary figures and images for: Retention and viral suppression in a cohort of HIV patients on antiretroviral therapy in Zambia: Regionally representative estimates using a multistage-sampling-based approach
Source: PLoS Med. 2019 May 31;16(5):e1002811. doi: 10.1371/journal.pmed.1002811 (PMC6544202; doi:10.1371/journal.pmed.1002811)

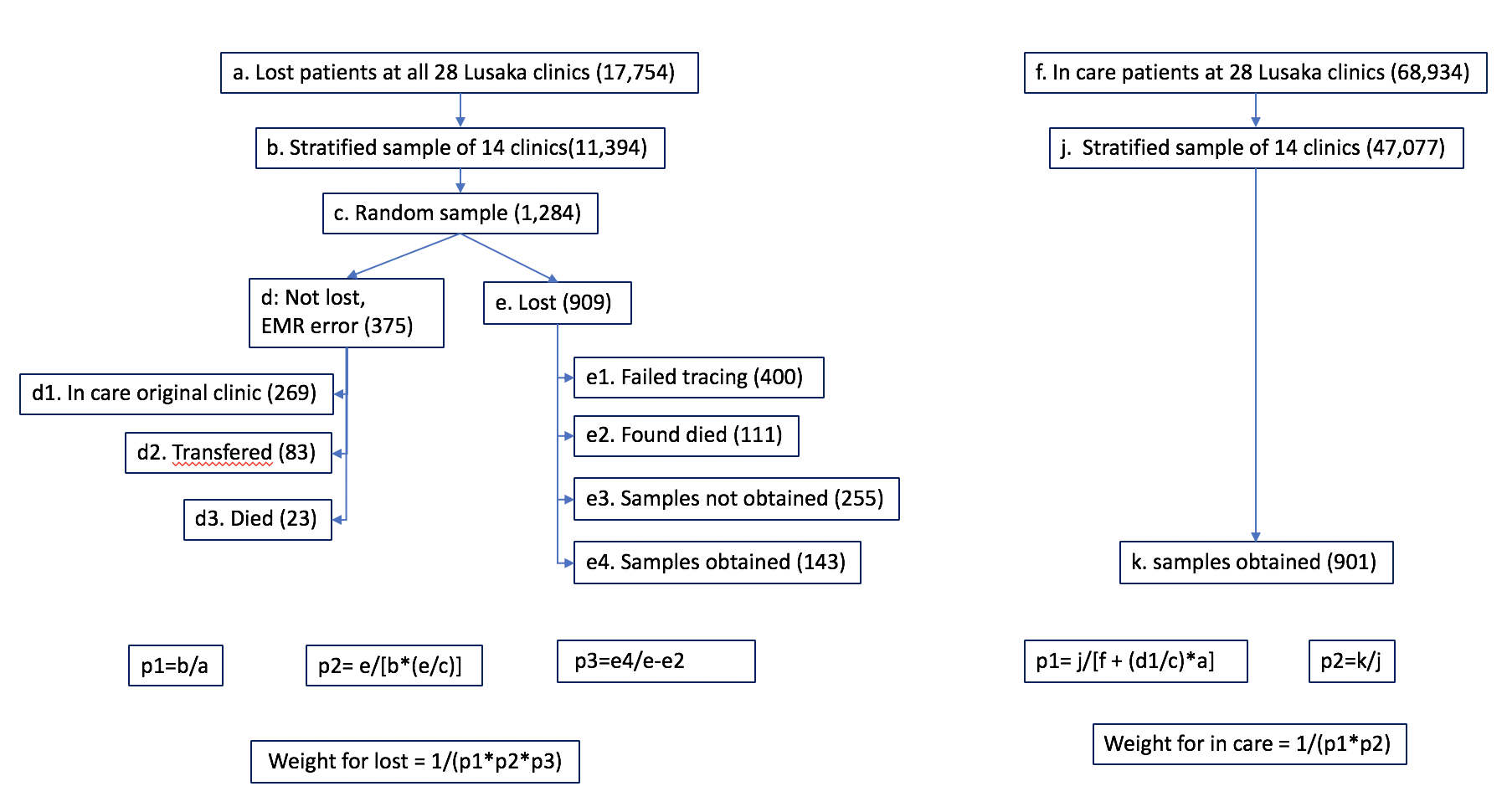

Supplement: S1 Fig — (TIF) [file pmed.1002811.s005.tif]

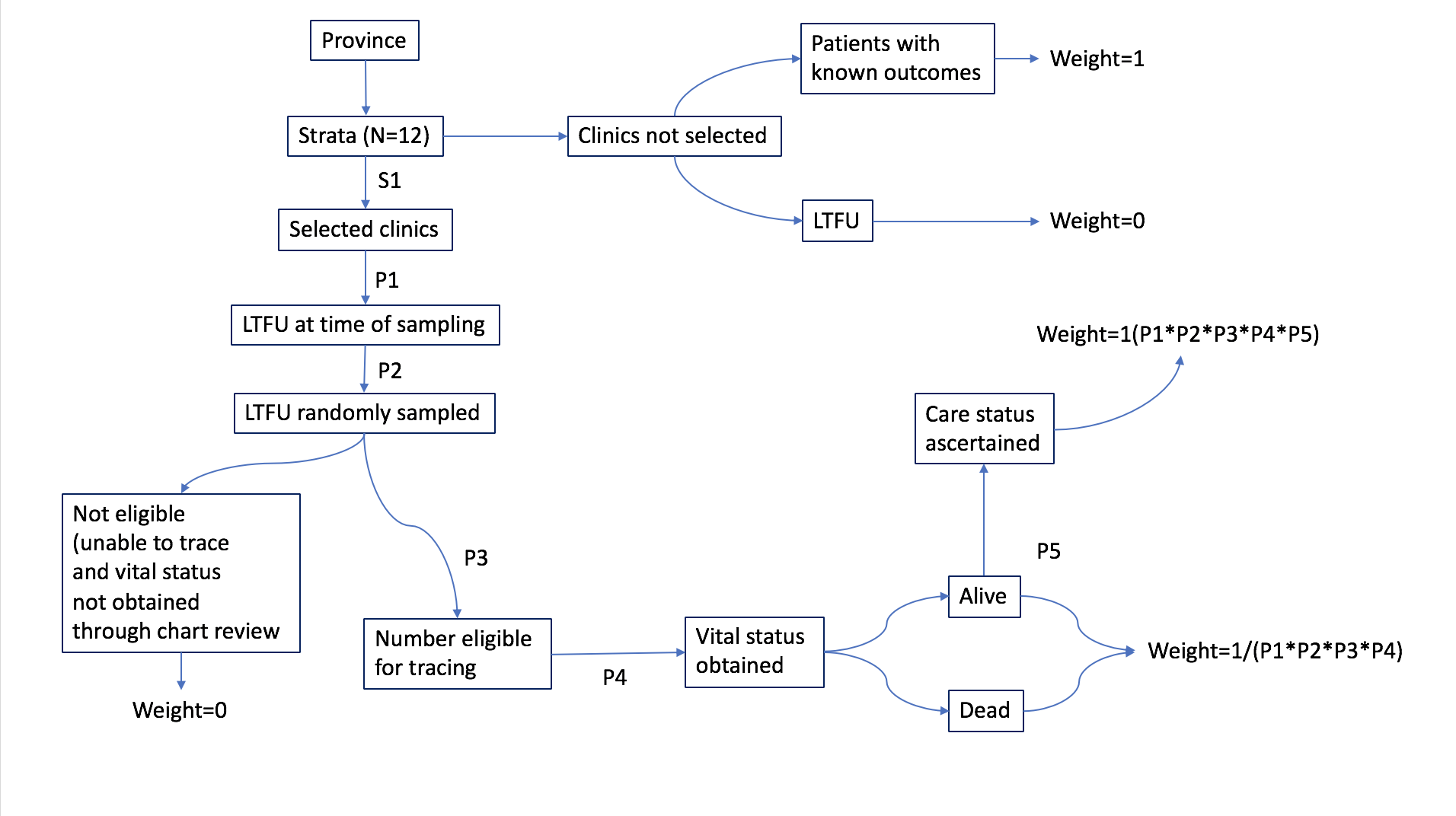

Supplement: S2 Fig — (TIF) [file pmed.1002811.s006.tif]

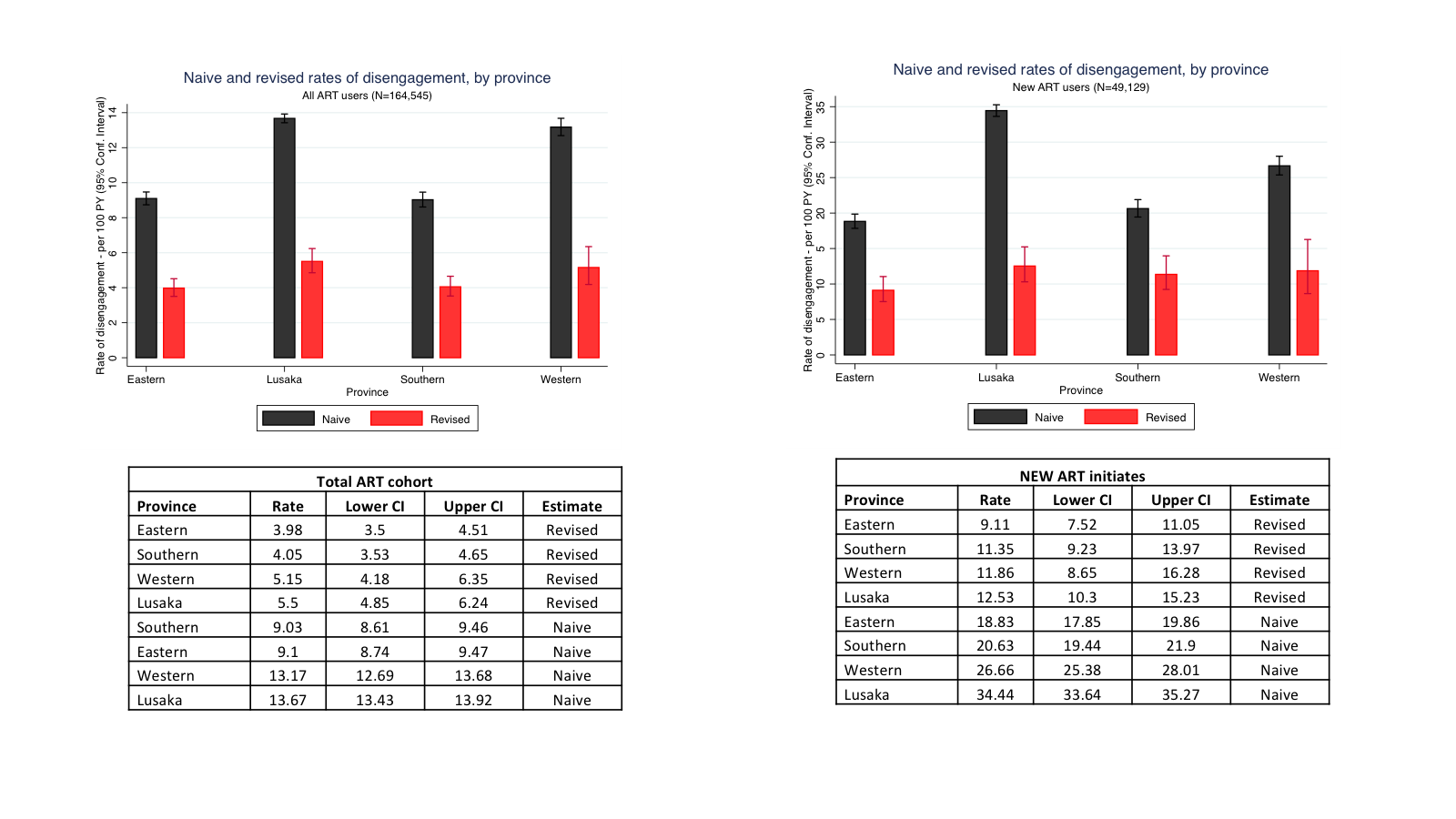

Supplement: S3 Fig — (TIFF) [file pmed.1002811.s007.tiff]
