# Supplementary material for: Retention and viral suppression in a cohort of HIV patients on antiretroviral therapy in Zambia: Regionally representative estimates using a multistage-sampling-based approach
Source: PLoS Med. 2019 May 31;16(5):e1002811. doi: 10.1371/journal.pmed.1002811 (PMC6544202; doi:10.1371/journal.pmed.1002811)
Supplement: S1 Table — (DOCX) [file pmed.1002811.s008.docx]

| **Variable** | | **Spearmans rho** | **p-value** |
| --- | --- | --- | --- |
| **Sex** | **Female** |  |  |
|  | **Male** | -0.0273 | 0.4235 |
| **Age (per 10 years)** |  | 0.0537 | 0.1155 |
| **Enrolment CD4 count**  **(per 50 cells/µl)** | **0-100** | -0.1075 | 0.0016 |
|  | **101-200** | -0.0381 | 0.2642 |
|  | **201-350** | 0.0037 | 0.9144 |
|  | **351-500** | 0.0068 | 0.8425 |
|  | **501 & >** |  |  |
| **WHO stage** | **1** | -0.0534 | 0.1176 |
|  | **2** | -0.0445 | 0.1925 |
|  | **3** | -0.0114 | 0.738 |
|  | **4** |  |  |
| **Province** | **Lusaka** | -0.0013 | 0.969 |
|  | **Eastern** | -0.02 | 0.5592 |
|  | **Southern** | -0.0526 | 0.1235 |
|  | **Western** |  |  |
| **Original facility type** | **Urban** | 0.0289 | 0.3977 |
|  | **Rural** | 0.0116 | 0.7336 |
|  | **Hospital** |  |  |
| **Facility size (per 1000 patients)** |  | 0.0173 | 0.6121 |
| **Marital status** | **Married** | 0.0169 | 0.6199 |
|  | **Single** |  |  |
|  | **Divorced** | 0.0212 | 0.5354 |
|  | **Widowed** | -0.0457 | 0.181 |
| **Disclosed** | **No** |  |  |
|  | **Yes** | -0.0164 | 0.6322 |
| **Education category** | **None** |  |  |
|  | **Lower-mid basic** | -0.031 | 0.364 |
|  | **Upper basic** | 0.0385 | 0.26 |
|  | **College** | -0.0093 | 0.7866 |

S1 Table: Test of proportional hazards assumption
