# Supplementary material for: Retention and viral suppression in a cohort of HIV patients on antiretroviral therapy in Zambia: Regionally representative estimates using a multistage-sampling-based approach
Source: PLoS Med. 2019 May 31;16(5):e1002811. doi: 10.1371/journal.pmed.1002811 (PMC6544202; doi:10.1371/journal.pmed.1002811)
Supplement: S2 Table — (DOCX) [file pmed.1002811.s009.docx]

S3 Table: Characteristics of new ART initiates

|  | | **Total New ART initiators** | **Lost** | **Sampled** | **Successfully traced** | **Alive** | **Known care status** |
| --- | --- | --- | --- | --- | --- | --- | --- |
| **Total participants** | | 49129 | 10217 | 993 | 734 | 560 | 483 |
| **Age at last visit (years)** | | 35 (29-42) | 33 (28-40) | 34 (28-41) | 34 (29-41) | 33 (28-40) |  |
| **Gender (male)** | | 18271 (37) | 3981 (39) | 397 (40) | 306 (42) | 214 (38) | 183 (38) |
| **Enrollment CD4 count (cells/µmol)** | | 281 (145-435) | 254 (125-409) | 268 (121-423) | 256 (119-412) | 295 (141-445) | 295 (134-450) |
| **Initiation CD4 count (cells/µmol)** | | 262 (138-388) | 238 (116-368) | 226 (102-364) | 225 (111-370) | 264 (130-395) | 257 (122-389) |
| **WHO stage at enrollment** | **Stage 1** | 23544 (48) | 4552 (45) | 433 (44) | 321 (44) | 273 (49) | 239 (49) |
|  | **Stage 2** | 8574 (17) | 1605 (16) | 174 (18) | 130 (18) | 88 (16) | 80 (17) |
|  | **Stage 3** | 8779 (18) | 2294 (22) | 180 (18) | 138 (19) | 93 (17) | 78 (16) |
|  | **Stage 4** | 777 (2) | 241 (2) | 32 (3) | 22 (3) | 10 (2) | 9 (2) |
|  | **Unknown** | 7455 (15) | 1525 (15) | 174 (18) | 123 (17) | 96 (17) | 77 (16) |
| **Province** | **Eastern** | 9234 (19) | 1163 (11) | 170 (17) | 136 (19) | 99 (18) | 93 (19) |
|  | **Lusaka** | 25644 (52) | 6672 (65) | 444 (45) | 305 (42) | 252 (45) | 213 (44) |
|  | **Southern** | 6564 (13) | 944 (9) | 196 (20) | 151 (21) | 108 (19) | 84 (17) |
|  | **Western** | 7687 (16) | 1438 (14) | 183 (18) | 142 (19) | 101 (18) | 93 (19) |
| **Year of enrollment** | **2004-2006** | 230 (0) | 58 (1) | 4 (0) | 2 (0) | 2 (0) | 1(0) |
|  | **2007-2009** | 1505 (3) | 328 (3) | 32(3) | 20 (3) | 18 (3) | 18 (4) |
|  | **2010-2012** | 5341 (11) | 1053 (10) | 109 (11) | 73 (10) | 59 (11) | 55 (11) |
|  | **2013-2015** | 42053 (86) | 8778 (86) | 848 (85) | 639 (87) | 481 (86) | 409 (85) |
| **Time on ART (days)** | | 225(63-407) | 49 (1-180) | 46 (1-170) | 59 (1-185) | 75 (1-213) | 88 (0-224) |
| **Disclosure** | **No** | 1293 (3) | 355 (3) | 26 (3) | 18 (2) | 11 (2) | 9 (2) |
|  | **Yes** | 43329 (86) | 8846 (87) | 871 (88) | 639 (87) | 486 (87) | 416 (86) |
|  | **Unknown** | 4507 (9) | 1016 (10) | 96 (10) | 77 (10) | 63 (11) | 58 (12) |
| **Education level** | **None** | 3100 (6) | 626 (6) | 90 (9) | 63 (9) | 43 (8) | 41 (8) |
|  | **Lower** | 14462 (29) | 2785 (27) | 263 (26) | 195 (27) | 136 (24) | 124 (26) |
|  | **Upper** | 19894 (40) | 4365 (43) | 392 (39) | 295 (40) | 236 (42) | 192 (40) |
|  | **College** | 1831 (4) | 374 (4) | 42 (4) | 35 (5) | 28 (5) | 23 (5) |
|  | **Unknown** | 9842 (20) | 2067 (20) | 206 (21) | 146 (20) | 117 (21) | 103 (21) |
| **Marital status** | **Single** | 5059 (10) | 1224 (12) | 135 (14) | 108 (15) | 87 (15) | 75 (15) |
|  | **Married** | 25988 (53) | 5281 (52) | 514 (52) | 386 (52) | 308 (55) | 270 (56) |
|  | **Divorced** | 5227 (11) | 1131 (11) | 93 (9) | 65 (9) | 41 (7) | 37 (8) |
|  | **Widowed** | 3397 (7) | 603 (6) | 64 (6) | 46 (6) | 26 (5) | 22 (5) |
|  | **Unknown** | 9458 (19) | 1,978 (19) | 187 (19) | 129 (18) | 98 (18) | 79 (16) |
| **Facility** | **Rural** | 5442 | 1198 (12) | 227 (23) | 189 (26) | 136 (24) | 120 (25) |
|  | **Urban** | 28053 | 6674 (65) | 540 (54) | 386 (53) | 315 (56) | 274 (57) |
|  | **Hospital** | 15634 | 2345 (23) | 226 (23) | 159 (22) | 109 (19) | 89 (18) |

Footnotes: (a) Missing for 12,683 (25,8%); (b) Missing for 9,588 (19,5%) (c) Lower = Lower to mid-basic schooling; Upper = Upper-basic or secondary school; College= College or university
