# Supplementary material for: Retention and viral suppression in a cohort of HIV patients on antiretroviral therapy in Zambia: Regionally representative estimates using a multistage-sampling-based approach
Source: PLoS Med. 2019 May 31;16(5):e1002811. doi: 10.1371/journal.pmed.1002811 (PMC6544202; doi:10.1371/journal.pmed.1002811)
Supplement: S3 Table — (DOCX) [file pmed.1002811.s010.docx]

| S3 Table (a): Naïve estimates (Total ART cohort) | | | | |
| --- | --- | --- | --- | --- |
| Care state | Days | Cumulative Proportion | Lower CI | Upper CI |
| ainc | 90 | 0.99 | 0.99 | 0.992 |
| tfo | 90 | 0.006 | 0.005 | 0.006 |
| Lost | 90 | 0.001 | 0.001 | 0.001 |
| Died | 90 | 0.003 | 0.002 | 0.003 |
| ainc | 180 | 0.946 | 0.944 | 0.947 |
| tfo | 180 | 0.011 | 0.011 | 0.012 |
| Lost | 180 | 0.039 | 0.038 | 0.04 |
| Died | 180 | 0.004 | 0.004 | 0.004 |
| ainc | 365 | 0.872 | 0.87 | 0.875 |
| tfo | 365 | 0.022 | 0.021 | 0.022 |
| Lost | 365 | 0.099 | 0.098 | 0.101 |
| Died | 365 | 0.007 | 0.006 | 0.007 |
| ainc | 545 | 0.806 | 0.803 | 0.81 |
| tfo | 545 | 0.031 | 0.03 | 0.032 |
| Lost | 545 | 0.154 | 0.152 | 0.156 |
| Died | 545 | 0.009 | 0.008 | 0.009 |
| ainc | 710 | 0.677 | 0.673 | 0.683 |
| tfo | 710 | 0.046 | 0.045 | 0.047 |
| Lost | 710 | 0.265 | 0.261 | 0.268 |
| Died | 710 | 0.012 | 0.011 | 0.012 |

| S3 Table (b): Revised estimates (Total ART cohort) | | | | |
| --- | --- | --- | --- | --- |
| Care state | Days | Cumulative Proportion | Lower CI | Upper CI |
| ainc | 90 | 0.977 | 0.977 | 0.98 |
| tfo | 90 | 0.014 | 0.013 | 0.014 |
| aooc | 90 | 0.001 | 0 | 0.001 |
| died | 90 | 0.008 | 0.007 | 0.008 |
| ainc | 180 | 0.958 | 0.957 | 0.961 |
| tfo | 180 | 0.022 | 0.021 | 0.023 |
| aooc | 180 | 0.007 | 0.006 | 0.007 |
| died | 180 | 0.013 | 0.012 | 0.013 |
| ainc | 365 | 0.918 | 0.916 | 0.92 |
| tfo | 365 | 0.043 | 0.042 | 0.044 |
| aooc | 365 | 0.015 | 0.015 | 0.016 |
| died | 365 | 0.024 | 0.023 | 0.024 |
| ainc | 545 | 0.844 | 0.84 | 0.847 |
| tfo | 545 | 0.093 | 0.092 | 0.095 |
| aooc | 545 | 0.026 | 0.025 | 0.027 |
| died | 545 | 0.037 | 0.036 | 0.038 |
| ainc | 710 | 0.765 | 0.76 | 0.769 |
| tfo | 710 | 0.147 | 0.145 | 0.149 |
| aooc | 710 | 0.039 | 0.038 | 0.041 |
| died | 710 | 0.049 | 0.048 | 0.05 |

**Ainc**: alive, incare original clinic; **tfo**: transferred to new clinic (official or self-transfer); **aooc**: alive, out of care - after tracing; **lost:** lost according to EMR; **died:** died according to EMR or after tracing
