# Supplementary material for: Retention and viral suppression in a cohort of HIV patients on antiretroviral therapy in Zambia: Regionally representative estimates using a multistage-sampling-based approach
Source: PLoS Med. 2019 May 31;16(5):e1002811. doi: 10.1371/journal.pmed.1002811 (PMC6544202; doi:10.1371/journal.pmed.1002811)
Supplement: S4 Table — (DOCX) [file pmed.1002811.s011.docx]

| S4 Table (a): Naïve estimates (New ART initiates) | | | | |
| --- | --- | --- | --- | --- |
| Care state | Days | Cumulative proportion | Lower CI | Upper CI |
| ainc | 90 | 0.977 | 0.975 | 0.979 |
| tfo | 90 | 0.012 | 0.011 | 0.013 |
| Lost | 90 | 0.003 | 0.003 | 0.004 |
| Died | 90 | 0.008 | 0.007 | 0.008 |
| ainc | 180 | 0.855 | 0.85 | 0.86 |
| tfo | 180 | 0.02 | 0.019 | 0.021 |
| Lost | 180 | 0.115 | 0.112 | 0.118 |
| Died | 180 | 0.01 | 0.009 | 0.011 |
| ainc | 365 | 0.709 | 0.701 | 0.717 |
| tfo | 365 | 0.036 | 0.034 | 0.038 |
| Lost | 365 | 0.241 | 0.236 | 0.246 |
| Died | 365 | 0.014 | 0.013 | 0.015 |
| ainc | 545 | 0.59 | 0.578 | 0.599 |
| tfo | 545 | 0.049 | 0.047 | 0.052 |
| Lost | 545 | 0.345 | 0.339 | 0.352 |
| Died | 545 | 0.016 | 0.015 | 0.018 |
| ainc | 710 | 0.359 | 0.318 | 0.399 |
| tfo | 710 | 0.068 | 0.062 | 0.075 |
| Lost | 710 | 0.551 | 0.522 | 0.579 |
| Died | 710 | 0.022 | 0.017 | 0.028 |

| S4 Table (b): Revised estimates (New ART initiates) | | | | |
| --- | --- | --- | --- | --- |
| Care state | Days | Cumulative proportion | Lower CI | Upper CI |
| ainc | 90 | 0.942 | 0.938 | 0.944 |
| tfo | 90 | 0.035 | 0.034 | 0.037 |
| aooc | 90 | 0.002 | 0.002 | 0.002 |
| died | 90 | 0.021 | 0.02 | 0.023 |
| ainc | 180 | 0.892 | 0.886 | 0.897 |
| tfo | 180 | 0.053 | 0.051 | 0.055 |
| aooc | 180 | 0.022 | 0.021 | 0.024 |
| died | 180 | 0.033 | 0.031 | 0.035 |
| ainc | 365 | 0.805 | 0.797 | 0.813 |
| tfo | 365 | 0.106 | 0.103 | 0.11 |
| aooc | 365 | 0.035 | 0.033 | 0.037 |
| died | 365 | 0.054 | 0.051 | 0.056 |
| ainc | 545 | 0.672 | 0.66 | 0.685 |
| tfo | 545 | 0.184 | 0.178 | 0.189 |
| aooc | 545 | 0.056 | 0.053 | 0.059 |
| died | 545 | 0.088 | 0.084 | 0.092 |
| ainc | 710 | 0.442 | 0.398 | 0.484 |
| tfo | 710 | 0.331 | 0.307 | 0.356 |
| aooc | 710 | 0.096 | 0.087 | 0.105 |
| died | 710 | 0.131 | 0.122 | 0.141 |

**Ainc**: alive, incare original clinic; **tfo**: transferred to new clinic (official or self-transfer); **aooc**: alive, out of care - after tracing; **lost:** lost according to EMR; **died:** died according to EMR or after tracing
