# Supplementary material for: Retention and viral suppression in a cohort of HIV patients on antiretroviral therapy in Zambia: Regionally representative estimates using a multistage-sampling-based approach
Source: PLoS Med. 2019 May 31;16(5):e1002811. doi: 10.1371/journal.pmed.1002811 (PMC6544202; doi:10.1371/journal.pmed.1002811)
Supplement: S5 Table — (DOCX) [file pmed.1002811.s012.docx]

S5 Table: Characteristics of patients eligible for viral load sampling

|  |  | **Received viral load** | | | | | **Eligible but no viral load obtained** | | | | |
| --- | --- | --- | --- | --- | --- | --- | --- | --- | --- | --- | --- |
|  |  | **N** | **%** | **Median** | **IQR** | | **N** | **%** | **Median** | **IQR** | |
| **Total participants** |  | 143 |  |  | Lower | Upper | 255 |  |  | Lower | Upper |
| **Age at last visit** |  |  |  | 34.9 | 30.1 | 41.0 |  |  | 36.5 | 30.1 | 41.6 |
| **Sex** | Female | 80 | 55.9 |  |  |  | 146 | 57.3 |  |  |  |
|  | Male | 63 | 44.1 |  |  |  | 109 | 42.7 |  |  |  |
| **Enrollment cd4 count** |  |  |  | 248 | 137 | 360 |  |  | 220 | 123 | 364 |
| **WHO stage at enrollment** | Stage 1 | 72 | 50.3 |  |  |  | 94 | 36.9 |  |  |  |
|  | Stage 2 | 19 | 13.3 |  |  |  | 46 | 18.0 |  |  |  |
|  | Stage 3 | 33 | 23.1 |  |  |  | 90 | 35.3 |  |  |  |
|  | Stage 4 | 9 | 6.3 |  |  |  | 6 | 2.4 |  |  |  |
|  | Unknown | 10 | 7.0 |  |  |  | 19 | 7.5 |  |  |  |
| **Year of enrollment** | 2004-2006 | 4 | 2.8 |  |  |  | 12 | 4.7 |  |  |  |
|  | 2007-2009 | 23 | 16.1 |  |  |  | 34 | 13.3 |  |  |  |
|  | 2010-2012 | 59 | 41.3 |  |  |  | 88 | 34.5 |  |  |  |
|  | 2013-2015 | 57 | 39.9 |  |  |  | 121 | 47.5 |  |  |  |
| **Year of ART initiation** | 2004-2006 | 3 | 2.1 |  |  |  | 9 | 3.5 |  |  |  |
|  | 2007-2009 | 21 | 14.7 |  |  |  | 31 | 12.2 |  |  |  |
|  | 2010-2012 | 46 | 32.2 |  |  |  | 70 | 27.5 |  |  |  |
|  | 2013-2015 | 73 | 51.0 |  |  |  | 145 | 56.9 |  |  |  |
| **Education level** | None | 3 | 2.1 |  |  |  | 10 | 3.9 |  |  |  |
|  | Lower-mid basic | 34 | 23.8 |  |  |  | 54 | 21.2 |  |  |  |
|  | Upperbasic/secondary | 77 | 53.8 |  |  |  | 138 | 54.1 |  |  |  |
|  | College/University | 10 | 7.0 |  |  |  | 16 | 6.3 |  |  |  |
|  | Unknown | 19 | 13.3 |  |  |  | 37 | 14.5 |  |  |  |
| **Marital status** | Single | 20 | 14.0 |  |  |  | 33 | 12.9 |  |  |  |
|  | Married | 76 | 53.1 |  |  |  | 138 | 54.1 |  |  |  |
|  | Divorced | 14 | 9.8 |  |  |  | 20 | 7.8 |  |  |  |
|  | Widowed | 10 | 7.0 |  |  |  | 21 | 8.2 |  |  |  |
|  | Unknown | 23 | 16.1 |  |  |  | 43 | 16.9 |  |  |  |
| **Facility** | Rural | 12 | 8.4 |  |  |  | 26 | 10.2 |  |  |  |
|  | Urban | 115 | 80.4 |  |  |  | 187 | 73.3 |  |  |  |
|  | Hospital | 16 | 11.2 |  |  |  | 42 | 16.5 |  |  |  |
| Time on ART |  |  |  | 511 | 135 | 1342 |  |  | 437 | 70 | 1187 |
